# Recent updates on COVID-19: A holistic review

**DOI:** 10.1016/j.heliyon.2020.e05706

**Published:** 2020-12-11

**Authors:** Shweta Jakhmola, Omkar Indari, Dharmendra Kashyap, Nidhi Varshney, Annu Rani, Charu Sonkar, Budhadev Baral, Sayantani Chatterjee, Ayan Das, Rajesh Kumar, Hem Chandra Jha

**Affiliations:** a Discipline of Biosciences and Biomedical Engineering, Indian Institute of Technology, Indore, India; b Discipline of Physics, Indian Institute of Technology, Indore, India

**Keywords:** Microbiology, Virology, Viruses, Epidemiology, Vaccines, Health disparity, Diagnostics, SARS-CoV-2, Mutations, Comorbidities, Signalling, Vaccination, Spike protein

## Abstract

Coronaviruses are large positive-sense RNA viruses with spike-like peplomers on their surface. The *Coronaviridae* family's strains infect different animals and are popularly associated with several outbreaks, namely SARS and MERS epidemic. COVID-19 is one such recent outbreak caused by SARS-CoV-2 identified first in Wuhan, China. COVID-19 was declared a pandemic by WHO on 11^th^ March 2020. Our review provides information covering various facets of the disease starting from its origin, transmission, mutations in the virus to pathophysiological changes in the host upon infection followed by diagnostics and possible therapeutics available to tackle the situation. We have highlighted the zoonotic origin of SARS-CoV-2, known to share 96.2% nucleotide similarity with bat coronavirus. Notably, several mutations in SARS-CoV-2 spike protein, nucleocapsid protein, PLpro, and ORF3a are reported across the globe. These mutations could alter the usual receptor binding function, fusion process with the host cell, virus replication, and the virus's assembly. Therefore, studying these mutations could help understand the virus's virulence properties and design suitable therapeutics. Moreover, the aggravated immune response to COVID-19 can be fatal. Hypertension, diabetes, and cardiovascular diseases are comorbidities substantially associated with SARS-CoV-2 infection. The review article discusses these aspects, stating the importance of various comorbidities in disease outcomes. Furthermore, medications' unavailability compels the clinicians to opt for atypical drugs like remdesivir, chloroquine, etc. The current diagnostics of COVID-19 include qRT-PCR, CT scan, serological tests, etc. We have described these aspects to expose the information to the scientific community and to accelerate the research.

## Teaser

Microbiology; Virology; Viruses; Epidemiology; Vaccines; Health Disparity; Diagnostics; SARS-CoV-2; Mutations; Comorbidities; signalling; Diagnostics; Vaccination; spike protein.

## 1 A brief introduction of coronaviruses and associated pandemics

In Latin, coronavirus (CoV) refers to "halo" or "crown" viruses as these have spike-like projections on its surface [1, 2]. The genome size of the virus ranges from 26 to 32 × 10^3^ bp and consists of multiple open reading frames (ORFs) (from 6 to 11). The first ORF constituting 67% of the whole virus genome is responsible for 16 non-structural proteins (nsps). The other necessary proteins are encoded by the remaining ORFs [3]. The four main structural proteins of the virus include the spike surface glycoprotein (S), envelope (E), membrane (M), and nucleocapsid (N). The S protein establishes the essential host cell tropism [4]. Further speaking of the first infectious CoV, the virus was isolated from birds with bronchitis in the 1930s [5]. CoVs comprises four genera: Alpha, Beta, Gamma, and Delta CoV. The first two genera of CoVs are known to infect humans, while the latter two predominantly infect birds [6]. In the 1960s, the first human coronavirus (HCoV) from the cultures of patients with a common cold was obtained [7]. Apart from the newly recognized severe acute respiratory syndrome coronavirus-2 (SARS-CoV-2), six other HCoVs have been identified, namely HCoV-NL63 and HCoV-229E, which belong to the genus Alpha CoV [8], HCoV-HKU1, HCoV-OC43, Middle East respiratory syndrome coronavirus (MERS-CoV), and SARS-CoV which are categorized as Beta CoVs [8].

CoVs are responsible for almost 30% of common colds, and often the individuals are infected with these viruses in their lifetime [9]. CoV infections display a seasonal pattern with an increased number of cases during winter and early spring [10]. Occasionally, the virus can infect other animals, apart from its natural host, and undergo mutations resulting in a more evolved virus capable of affecting a broader and distinct population. CoVs were mainly highlighted after the 2003 SARS pandemic followed by the MERS epidemic in 2012, and now the very recent SARS-CoV-2 outbreak [11]. The 2002–2003 SARS pandemic affected almost 8,096 and claimed 774 lives of four continents before its containment [12]. The SARS CoV resembled the bat CoV, its natural host [13]. However, the virus was investigated to spread to humans by handling and consuming palm civets, raccoon dogs, and Chinese ferret badgers sold and slaughtered at the wet markets of China [13]. The virus infected individuals with severe pulmonary syndrome and showed a mortality rate of 10% [14]. Furthermore, the SARS-CoV S protein receptor-binding domain (RBD) binds to the host receptor angiotensin-converting enzyme2 (ACE2) [15] with CD209L as a substituting receptor [16]. After SARS-CoV, another highly pathogenic CoV outbreak was the MERS-CoV pandemic [17] that mostly affected the Arabian Peninsula [18]. MERS-CoV lasted for seven years, even after its spread in the confined areas. To date, almost 2500 cases of MERS have been confirmed with a mortality rate of ~35% [19]. It is more closely associated with bat CoVs than that of other HCoVs [20]. For entry, MERS-CoV uses dipeptidyl peptidase 4 (DPP4, also known as CD26) as a receptor [21, 22]. To date, no vaccination or particular treatment is functional for MERS-CoV; however, a recent candidate DNA vaccine developed from MERS-CoV S protein subunit 1 (S1) is under study [23]. MERS-CoV has shown recurrence in the human population by direct or indirect contact with infected primary reservoirs, which are the dromedary camels [24].

The severe pathogenic ability of SARS viruses to cross-species and undergo high mutation rates has resulted in the emergence of a new virus, i.e., SARS-CoV-2. The virus originated in the Wuhan province of China, and the disease, coronavirus disease-2019 (COVID-19), has become an emerging global health emergency since 11^th^ March 2020 [25]. The virus affected a considerable population within a short period indicating its rapid potential to spread. The virus's ability to infect humans was reported due to the evolution of RBD towards ACE2 receptors expressed on various human cells [21]. The virus is known to reach and infect different organs like the brain, kidney, liver, gastrointestinal system, etc. thereby causing multiple organs failure [26, 27, 28]. Symptoms of COVID-19 that resemble SARS and MERS include fever, cough, and shortness of breath [29]. Furthermore, SARS-CoV usually infects the young population, and MERS-CoV affects people aged above 50 years. However, SARS-CoV-2 is mostly known to cause severe manifestations in middle-aged and older people [30, 31]. Collectively, our review aims to provide information on SARS-CoV-2 evolution, associated comorbidities, and its influence on infected individuals at the molecular and pathophysiological levels. Briefly, we also highlight the probable vaccine candidates from varied organizations in different phases of clinical trials. In addition, a glimpse into the diagnostics, along with possible interventions adapted to date to contain the virus, is presented. The information about various facets of SARS-CoV-2 infection is scattered in numerous types of reports. A comprehensive presentation of updated information is therefore necessary. The information here will enable us to understand SARS-CoV-2 from different angles and aid in the research of COVID-19.

## 2 Zoonotic origin of SARS-CoV-2

Tracing back the tracks of history and early epidemiological studies, it has become evident that HCoV infections have zoonotic origins [32]. The intermediate host, to which the virus is recently introduced, can amicably serve as a zoonotic source with the capability to transfer the infection to humans at a full scale. The intermediate host provides the virus with the necessary conditions to amplify and replicate transiently [33]. Besides, the virus can come to a dead-end situation if its sustenance is supported within the intermediate host or evolve itself according to the host, making it its natural reservoir host. What needs to be investigated regarding SARS-CoV-2 is if the virus acclimatizes to humans and can transmit without its intermediate host. Therefore, identifying the animal source can provide us with preventive human disease interventions. Phylogenetic studies have shown bats and rodents as the reservoir of most alpha- and beta-CoVs evident [34]. For long, the CoVs are known to cross the barrier of species specificity and enter the human population like MERS-CoV, SARS-CoV, and SARS-CoV-2 [35].

Homology studies have demonstrated 96.2% nucleotide similarity between the SARS-CoV-2 and RaTG13, a bat CoV obtained from *Rhinolophus Affinis* [36]. Based on similar reviews, another conclusion was drawn that bats cannot serve as intermediate reservoir hosts for SARS-CoV-2 until an almost identical CoV is acquired from bats. The spread of the virus has to be from some of the wild animals dealt at the Huanan seafood wholesale. The site accounts for the initial cases of COVID-19 and seems suitable for an animal to human transmission [37]. Further, the metagenomic studies hint towards pangolins (*Manis javanica*) as a plausible intermediate host capable of harboring beta-CoVs related to SARS-CoV-2 [38]. Jaimes *et al.* performed S protein-based phylogenetic analysis of pangolin-CoVs from Malayan pangolins brought into China from Guangxi and Guangdong Province, SARS-CoV-2, BatCoV-RaTG13, and other beta coronaviruses [39]. Also, a detailed similarity analysis of different betacoronavirus involved 148 human SARS-CoV-2 sequences, six pangolin CoVs (PcoV_GX_P5L, P2V, P4L, P1E, P5E, and MP789), and two bat CoVs namely RaTG13 (from *Rhinolophus affinis*) and RmYN02 (from *R. malayanus*) [40]. The CoV (MP789 CoV) derived from these small endangered mammals share nucleotide homology, up to 85–92%, with the SARS-CoV-2 [38,40]. Surprisingly, despite the higher sequence homology of SARS-CoV-2 with RaTG13 (96.3%), the SARS-CoV-2 RBD shares 97.4% amino acid sequence similarity with RBD of Guangdong pangolin CoV [38].

Supporting the link between the pangolins and SARS-CoV-2, others report the presence of similar viral fragments from diseased pangolins lung samples [41]. Although no direct evidence exists that defines pangolins as the origin of SARS-CoV-2. Theories also suggest the chances of a highly occurring event in CoVs called recombination between the above two species in a third animal species [42]. Several earlier examples demonstrate the ability of the CoVs to be able to transmit from domestic animals to humans; for instance, the HCoV-OC43 pandemic around 1890 was recorded after the virus jumped from domestic livestock to humans [43]. Bats are apt agents for interspecies virus dispersal because of many reasons like their ability to be in close social densely packed colonies, longevity, and ability to fly, which in turn allows them to cover and spread the pathogen to wider areas [44]. Usually, bats transmit the microorganisms through intermediate hosts, mostly when the intermediate host consumes the partially digested bat food, which serves as a potential source of infection [44]. These placental mammals provide suitable body temperature, allowing replication of viruses sensitive to increased temperatures [45]. Bats have long been known to serve as reservoirs of various microorganisms, especially RNA viruses, implicated in several human diseases. It is noteworthy to mention that more than 200 viruses are associated with bats, including SARS and MERS coronaviruses, as well as Ebola and Marburg viruses [45].

CoVs being RNA viruses, have an immense potential to introduce mutation during replicating its genome, with ~10^−4^ average substitutions per site in a year [46]. The mutation rates give the viruses the ability for interspecies co-evolution. Further, the virus family has a proofreading exoribonuclease, elimination of which can result in an even higher mutation rate [47]. Nevertheless, the mutation rate is approximately a million times more in the CoVs rather than their hosts [48]. However, the rate of variation in SARS-CoV-2 is relatively lower to SARS-CoV, therefore making it more adaptable to humans [49]. The CoVs have an ample number of RNA fragments (ORFs), and the viruses often switch the templates during replication. Notably, it is known that new CoVs could generate homologous recombination of full length and subgenomic RNAs [50]. An example of this is natural recombination between HCoV-HKU1 and HCoV-OC43 [51].

Additionally, the host plays a crucial role in the interspecies spread of the virus. In other words, a host may be suitable for infection by one virus, with just a difference of a few amino acids substitution in its protein. A cryo-electron microscopy study demonstrates that a difference of 30% in between the S protein of SARS-CoV-2 and SARS-CoV is responsible for 20 fold higher affinity of the SARS-CoV-2 S protein with ACE2 receptors expressed by the human cells [52]. Therefore, these variations could be responsible for establishing a CoV in humans upon transmission from their natural reservoir host. Nonetheless, the HCoVs frequently possess factors that subvert the host restriction factors for successful interspecies transmission.

## 3 Mutations in SARS-CoV-2 proteins from various isolates around the world

**Figure 1:**
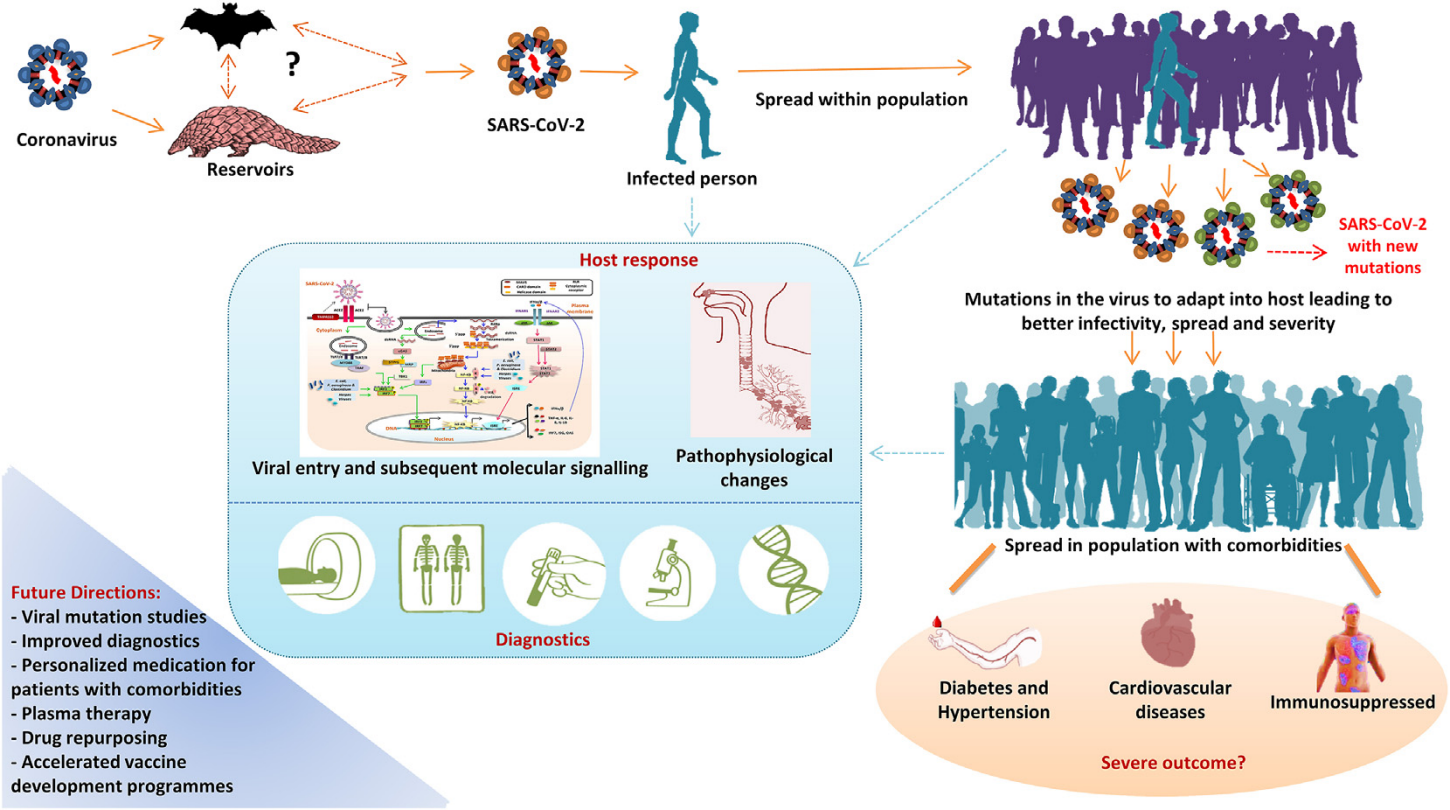
**Overview of various aspects associated with SARS-CoV-2 infection: Origin, transmission, risk factors within a population, host response, diagnostics, and respective future directions.** SARS-CoV-2 has a zoonotic origin. It might have travelled to humans from bat through an intermediate host like a pangolin. During further spread among the human population, it has undergone many mutations. Additionally, individuals with comorbidities may be susceptible to infection or disease severity. Viral entry inside the cell with subsequent molecular signaling and pathophysiological changes within the host is needed to be understood for better diagnosis and therapeutic targets. However, various diagnostics tools have made detection of infection easier. In the near future, the current pandemic demands more viral mutation studies, the development of better diagnostic tools, accelerated research for vaccine development, drug repurposing, and further amendments in the treatment of patients with comorbidities.

The potency of viruses to adapt to new hosts and niches is based on their ability to generate beneficial genomic diversity in short periods [53]. Nucleotides substitutions have been determined as one of the critical mechanisms of viral development [54]. These substitutions not only alter the genome of the virus but also get mirrored into the subsequent proteome. The changes in protein sequences can cause anomalies in their structure and function, giving the virus new tools to adapt and survive. As mentioned previously, the RNA viruses have a huge potential to mutate. Likewise, SARS-CoV-2, during its spread in the different populations in varied subcontinental areas, has undergone distinct mutations, whereas very few mutations have been observed at the geographical level [55, 56, 57]. This further adds up to the challenges in fundamental research regarding SARS-CoV-2 as well as in vaccine and drug development (Figure 1).

We have briefly reviewed several mutations reported in various SARS-CoV-2 proteins from different sub-continental areas. The open-reading frames (ORFs) in the SARS-CoV-2 genome are similar to those of all β-coronaviruses [58, 59, 60]. ORF1a/b encodes various essential accessory proteins like proteases, helicase, etc. The S, E, M, and the N proteins are encoded by their distinct ORFs. Some other ORFs like ORF3a, ORF6a, ORFF7a, and ORF8a, are involved in different putative functions such as inhibition of interferon-β (IFN-β), DNA synthesis, etc. as summarized in a report [61]. Nonetheless, the function of ORF9b and ORF10a is yet to be explored [59, 61]. In a report addressing mutations in different ORFs, the ORFs of E protein, M glycoprotein, ORF6, ORF7b, and ORF10 were found to be conserved while several mutations were observed in ORF1a, ORF1b, ORF3a, ORF7a, ORF8, as well as in N and S protein ORF [62]. Another study on Indian isolates observed that the 5′terminal containing the genes for the ORF1ab, S, ORF3a, and E are more prone to mutations than 3′terminal, and are key drivers for virus diversity [56].

It is noteworthy that most of the worldwide reported mutations were from the S protein ORF [63, 64, 65]. The S protein mutations alter the receptor binding affinity, interaction, and amalgamation of viral covering with host cell membranes, thereby amending virus-host interaction. One of S protein mutations reported was D614G, which lies at the S1/S2 subunit junction [63]. The mutation is reported in the isolates from Chile, Egypt, Germany, India, and the USA (Table 1). D614G mutation in S protein along with other mutations, result in significantly more infectious virus particles and provide fitness advantages [57, 66]. Moreover, pseudoviruses with alone D614G mutations have shown increased infectious titers compared to the non-mutated forms [67, 68]. Other observed S protein mutations include R407I, A930V, and G1124V from Indian isolates and Q57H and V367 from French isolates [63, 64, 65]. These mutations could alter the infectivity, host-dependent modifications, replication cycle, evolutionary adaptation, species recognition, host receptor affinity, and pathophysiology of the disease [55, 65, 66, 67]. L84S, G392D, and G251V were also reported from the USA, German and Brazilian isolates, respectively [63]. Additionally, some of the mutations such as A475V, L452R, V483A, and F490L in RBD of S protein make SARS-CoV-2 resistant against neutralizing antibodies and are less infectious [66]. RBD mutation enhances the virus's binding affinity with the ACE2 receptor; hence, it promotes the replication and infectivity of the virus [56].

**Table 1:** Comprehensive view of various mutations in SARS-CoV-2 proteins (S protein, N protein, PLpro and ORF3a) among different country-wise isolates.

Countries	Mutations														
	S protein									N protein			RdRp	PLPro	ORF3a
	Q 57 H	L 84 S	G 251 V	V 367 F	G 392 D	R 407 I	D 614 G	A 930 V	G 1124 V	S 194 L	R 203 K	G 204 R	P 323 L	A 225 V	G 5214 S
Brazil			+												
Chile							+			+	+	+	+	+	
Egypt							+								+
France	+			+											
Germany					+		+								
India						+	+	+	+		+	+	+		
USA		+					+			+					
REF	a)	a)	a)	b)	a)	c)	a)	c)	d)	b)	e)	f)	e)	e)	g)

[References: a) [63]; b) [64]; c) [76]; d) [69] e) [70]; f) [71]; g) [75]].

N protein is yet another critical protein playing structural as well as non-structural roles [68]. This protein also has strong immunogenicity [68]. Two reported mutations, R203K and G204R, have been found in isolates from Chile and India, which trigger the viral capsid formation [69, 70, 71]. In addition, S197L mutation in N protein is reported in isolates from Chile and the USA [64]. These mutations could modulate the interaction, the protein functionality, and the structural integrity for better organization in viral assembly [69, 72]. Furthermore, the viral RNA dependent RNA polymerase (RdRp) plays a vital viral genome replication task. P323L mutation is reported in the isolates from Chile and India [70]. This mutation's significance needs to be investigated and could provide insights into the SARS-CoV-2 evolution and the role of this enzyme in the current mutation rate. Mutations are also reported in the papain-like protease (PLpro), an enzyme that performs the cleavage of replicase polyprotein at conserved sites. This step is necessary for the development of functional replication assembly [73]. The A225V mutation in this protein was found in isolates from Chile [74]. Additionally, ORF3a is an accessory protein of SARS-CoV-2 and is known to induce pro-apoptotic activity [74]. The G5214S mutation is observed in ORF3a of the isolates from Egypt [75]. However, the contribution of each of these mutations in terms of SARS-CoV-2 evolution, virulence, and transmission needs to be investigated further.

## 4 Comorbidities and its alliance with SARS-CoV-2

Emanating data speculates elevated association and fierce mortality rate in COVID-19 patients with comorbidities [77]. We have briefed about some of the COVID-19 associated comorbidities.

### 4.1 Diabetes and hypertension: an inevitable risk factor for COVID-19

The involvement of hypertension and diabetes in COVID-19 patients is not surprising, given the elevating myriad of both of these chronic diseases globally [78, 79, 80, 81, 82, 83]. Diabetes and hypertension are interrelated; reports suggest that hypertension is twice as common in diabetic patients as in non-diabetics [84, 85]. This overlap generally causes ischemic cerebrovascular disease, retinopathy, diabetic nephropathy, and sexual dysfunction [80]. Diabetes alone can lead to severe cardiovascular diseases and an increase in vascular smooth muscle cells [85]. Various meta-analyses and other independent studies have revealed hypertension and diabetes to be among the most prevalent comorbidities associated with COVID-19 [86,87,88,89]. Recently we have analyzed the data of hospitalized and deceased COVID-19 patients from various countries with comorbidities like diabetes, hypertension, heart diseases, immunocompromised and neurological diseases. The report suggests that comorbidities particularly, diabetes, hypertension, and heart diseases, are widely related to deaths in COVID-19 patients [77]. Reports indicate that out of the total COVID-19 patients with at least one comorbidity, 28–30% had hypertension, and 12–19% had diabetes [90, 91]. Moreover, diabetic COVID-19 patients exhibit an increased risk of thrombotic complications [92]. Reports suggest the association of type 2 and type 1 diabetes with COVID-19 [93]. SARS-CoV-2 infection can trigger severe metabolic complications in individuals with pre-diabetic conditions and influence diabetic ketoacidosis and hyperosmolarity. Also, it could lead to the onset of diabetes [93, 94].

Insulin resistance in diabetic patients leads to sodium retention, stimulation of the sympathetic nervous system, and the renin-angiotensin system [95, 96]. However, as the ACE2 has a protective role in blood pressure, its deficiency causes hypertension [97, 98, 99]. Diabetes and hypertension patients are prescribed with ACE inhibitors (ACEIs) and angiotensin II receptor blockers (ARBs) [100]. Upon use of ACEIs or ARBs, to compensate ACE inhibition, ACE2 overexpression is facilitated, hence increasing patients' vulnerability [101, 102]. ACE2 can also be elevated by thiazolidinediones and ibuprofen [100]. Few studies recommend that due to the finite availability of serine protease TMPRSS2, increased ACE2 expression does not result in more viral entry. TMPRSS2 inhibitor camostat mesylate has been demonstrated to obstruct SARS-CoV-2 entry [88, 103].

### 4.2 Cardiovascular diseases: a scary association with SARS-CoV-2 infection

Cardiac conditions like arrhythmia, cardiomyopathy, and coronary heart disease are significant cardiovascular comorbidities seen in severe COVID-19 cases [104]. Earlier reports have demonstrated the presence of the virus in heart autopsy samples. In the case of heart tissue, SARS-CoV-2 receptors are highly expressed [105]. Moreover, in mice, the virus led to ACE2 dependent myocardial infections [106]. The SARS-CoV and SARS-CoV-2 show similarities in their S protein, more specifically in the RBD domain. Hence it has been hypothesized that SARS-CoV-2 could infect cardiac tissue and aid in the severity of the disease [107]. Another potent HCoV, MERS-CoV, has also been associated with acute myocarditis and heart failure [108]. SARS-CoV-2 shares similar pathogenicity as MERS-CoV; therefore, SARS-CoV-2 can increase the disease complexity in the patients. Furthermore, SARS-CoV-2 infection injures the myocardium, which is determined by an increase in levels of myocardial biomarkers like creatine kinase (CK), creatine kinase MB isoenzyme (CK-MB), and lactate dehydrogenase (LDH) [107, 109, 110]. The specific biomarker with high-sensitivity is cardiac troponin I (hs-cTnI). However, the biomarkers do not always alter in the same pattern [110]. According to a study among 187 COVID-19 patients, 27.8% exhibited myocardial injury, demonstrated by elevation of troponin (TnT) levels. The study suggested patients with underlying cardiovascular diseases and escalation of TnT levels had the highest mortality (69.44%) [111]. Plasma TnT levels were positively correlated with plasma high-sensitivity C-reactive protein levels, showing possible myocardial injury association with inflammatory pathogenesis during the disease progression [111].

Importantly, severe COVID-19 patients with cardiovascular problems have difficulty in gas exchange and might lead to hypoxemia [112]. Thus, the virus infection triggers an exaggerated immune response by releasing interleukin-2 (IL-2), IL-6, IL-10, granulocyte colony-stimulating factor (GCSF), interferon-γ (IFN-γ), monocyte chemoattractant protein-1 (MCP-1), macrophage inflammatory protein-1-alpha (MIP-1-α), and tumor necrosis factor-α (TNF-α) mediators. It leads to cytokine storms and contributes to myocardial tissue inflammation, ultimately leading to heart dysfunction [113]. The excessive release of pro-inflammatory cytokines further triggers a reduction in coronary blood flow, decreases in oxygen supply, destabilization of coronary plaque, and micro-thrombogenesis [114]. Also it has been known that ACEIs/ARBs upregulated ACE2 which can make cardiac tissue more susceptible to SARS-CoV-2 infection [115]. It leads to an increase in severity in cardiovascular patients.

### 4.3 Does SARS-CoV-2 infections in immunosuppressive conditions worsen the outcome?

An immunocompromised state, co-pathogen, and host factors are correlated with increased risk for respiratory diseases [116]. The widespread COVID-19 has put forward a stringent situation to work on the treatment of critically ill patients [117]. According to a study, the chance of acquiring HCoV-infection in immunocompromised patients compared to healthy controls was twice as likely [118]. However, a report of SARS-CoV infection in AIDS patients suggests mild SARS infection because highly active antiretroviral therapy (HAART) might have benefited in fighting viral factors [119]. In such cases, the symptoms of SARS-CoV infection in the AIDS patient may not be noticed, and hence the patient may serve as a carrier in spreading the virus. Additionally, an animal model study performed on Syrian golden hamsters regarding immunocompromised state and SARS-CoV infection, little clinical illness, and no mortality were observed after virus infection [120].

According to an Italian report on patients with chronic arthritis treated with disease-modifying antirheumatic drugs (DMARDs), the patients do not seem to be at increased risk of respiratory or life-threatening complications from SARS-CoV-2 compared to the general population [121]. Few reports suggest that the most recently approved antirheumatic drugs prove to be strong allies in the fight against COVID-19 as they can precisely target the critical steps of the immune response that became dysregulated during the disorder. Due to this fact, the SARS-related lung damage can be caused more by an exaggerated immune response than the virus itself [122].

Respiratory infections may be present in solid organ transplant (SOT) patients that are under continual immunosuppression. After the treatment with a minimized immunosuppressant dose, these patients recovered successfully [123, 124]. A recent study from Italy correlated the occurrence of SARS-CoV-2 in patients undergoing doctoral investigation of SOT, cirrhosis, autoimmune liver disease, chemotherapy for hepatoblastoma [125]. Even after being positive for SARS-CoV-2, the patients did not develop acute lung pathology of SARS. A study from Spain regarding COVID-19 in kidney transplant patients and another from China related to heart transplant patients suggest that there could be an atypical presentation of symptoms (fever, diarrhea, fatigue) instead of typical SARS symptoms [126, 127]. These reports indicate the importance of careful monitoring and vigilant follow up of the patients with SOT during the COVID-19 outbreak [128].

Immunocompromised individuals have a similar threat of COVID-19 as that of others. Whereas, the outcome variations can depends upon an individual's immune status. A vigilant follow up for any symptoms is needed as there could be atypical symptoms representation. The immunosuppressive drug regime has to be adjusted as per the case based on the patient's clinical condition, age, gender, and previous history.

## 5 Lungs pathophysiology: the drive of SARS-CoV-2 inside the body

SARS-CoV-2 fabricates the complexities of the respiratory tract [129]. SARS-CoV-2 could transmit among people through respiratory droplets and contact routes [130]. Droplet transmission occurs when a person comes in close contact (~1 m) with an infected patient. During this, a healthy individual's mouth, nose, and eyes get exposed to potentially infective respiratory droplets [131]. Infection feasibility [132] also gets transmitted through fomites in the close environment of an infected person [133, 134]. The virus further attacks an individual's exposed part's epithelial lining, gaining entry in the host tissue. Once in the cell, it hijacks the cellular machinery producing multiple copies. Further, it could reach the lungs through the respiratory tract [134]. Additionally, in the lungs, the virus triggers morphological as well as biochemical alterations.

Post SARS-CoV-2 infection, the histopathological changes in different body organs, including lungs, kidneys, gastrointestinal tract, liver, heart, skin, and brain, are observed [135]. The lung biopsy examination of COVID-19 patients shows dispersed alveolar damage, patches of hemorrhagic necrosis, alveolitis with atrophy, intra-alveolar fibrinous exudate, loose interstitial fibrosis [135, 136]. There is diffuse alveolar damage (DAD), vascular congestion with occasional inflammatory cells, damaged pneumocytes with focal sloughing and formation of syncytial giant cells, etc. Additional findings include intra-alveolar hemorrhages, type II pneumocyte hyperplasia, fibrinoid necrosis of the small vasculature, and abundant intra-alveolar neutrophil infiltration to bronchopneumonia [135]. Most of the histopathological findings are similar to those described in SARS-CoV and MERS-CoV.

The radiological findings include bilateral and peripheral ground glass-like opacification in COVID-19, also observed in MERS-CoV infection [137, 138]. As the SARS-CoV-2 disease progresses, ground-glass opacification decreases, and consolidation with mixed patterns and subsequent resolution of air space changes is observed [137]. In contrast, intra-alveolar plugs have been observed in both SARS-CoV and SARS-CoV-2 infections, whereas multinucleated pneumocytes disperse alveolar damage, tissue plug formation demonstrated in SARS-CoV infection [138, 139]. Hence, the radiological findings have few limitations like the formation of multinuclear pneumocytes are not only found in the case of SARS infection, but also seen in the case of other viral diseases like *Paramyxoviridae* family viruses, measles, mumps, RSV, parainfluenza viruses, and metapneumovirus, etc. [140].

The damage of lung tissues in COVID-19 patients might be an outcome of inflammatory responses like cytokines/chemokine storm [26, 141]. The pro-inflammatory mediator's level increases over anti-inflammatory, which results in changes in the biochemical territory of lung tissues [142]. The SARS-CoV-2 infection possibly stimulates the release of interleukin-1β (IL-1β), IFN-γ, interferon-inducible protein-10 (IP-10), and MCP1 (a pro-inflammatory cytokine) by triggering activation of T-helper-1 (Th1) cell responses [141]. In the case of SARS-CoV patients, lung damage gets deteriorated with an increasing amount of IL-1β, IL-6, IL-12, IFN-γ, IP-10, and MCP1 whereas, in MERS-CoV infection, IFN-γ, TNF-α, IL-15, and IL-17 set off inflammation in the lungs [49, 143]. The interferon-α/β (IFN-α/β), chemokine (C–C motif) ligand 5 (CCL5) genes also get activated in these viral infections [144]. Additionally, a few factors which deteriorate the respiratory system, like asthma, smoking where lungs already have prior exposure to inflammatory reactions, might be more susceptible to SARS-CoV-2 infection [145]. In asthma, the lungs' epithelial lining triggers an antiviral response that relies on the prompt induction of cytokines, mostly type I/III- interferons (IFNs), and the type-2 cytokines like IL-13, IL-4, IL-5, IL-9, etc. These cytokines ultimately weaken the epithelium barrier and commence pro-inflammatory reactions [121, 144, 145]. Interestingly, few reports have observed that smoking upregulates the ACE2 level in airways epithelium [146, 147]. Smoking not only accelerates phosphorylation of microtubule-associated protein kinase (MAPK) while also the production of specific cytokines, such as type I and III IFNs, IL-1β, IL-18, TNF-α, IL-6, IL-8, and the "alarmin" IL-33, which lead to the inflammation in lungs [148, 149]. Apart from ACE2, SARS-CoV-2 takes entry inside the cells by using receptors like CD147 and targets key signaling molecules like nuclear factor kappa light chain enhancer of activated B cells (NK-kB), interferon regulatory factors (IRFs), IRF-3, and IRF-7, which ultimately create pathophysiological conditions in the lungs [150]. Further, we have discussed these factors in greater detail in the molecular signaling part.

## 6 Molecular signaling involved in the development of pathophysiology of SARS-CoV-2 infection

**Figure 2:**
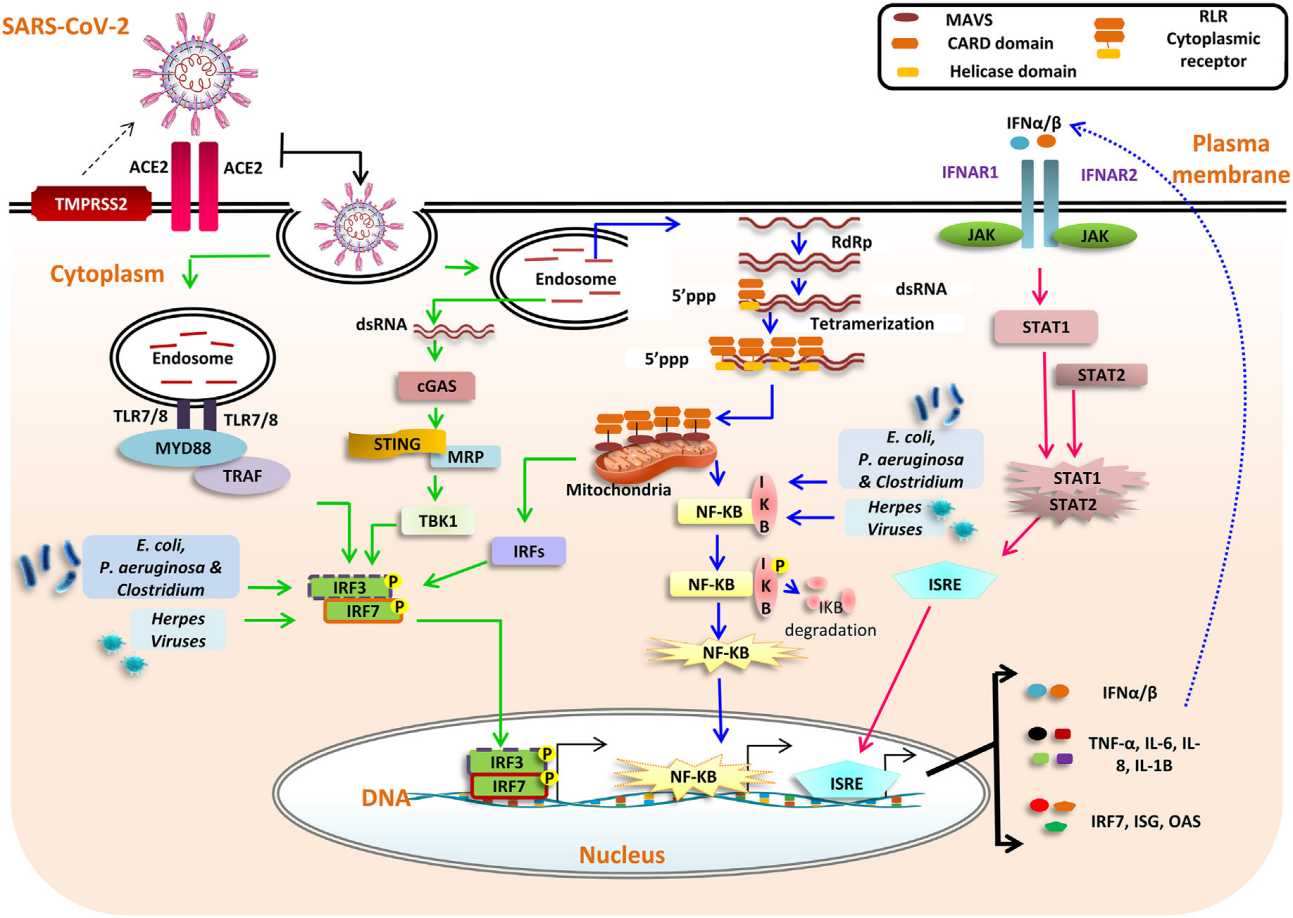
**Illustration of molecular signaling involved in SARS-CoV-2 infection.** SARS-CoV-2 spike protein is detected via ACE2 with the help of TMPRSS2 protease and initiates phagocytosis into the cell. TLRs, RLRs, and cGAS detect viral RNA in the endosomal and cytosolic compartments. TLR7/8 activates type 1 Interferon genes (IFN α/β) via IRF3 and IRF7 dimerization. The RLRs cytosolic receptors detect dsRNA, which is converted from ssRNA by the action of RNA dependent RNA polymerase (RdRp) after hijacking the host ribosomal machinery. Further, RLRs trigger NF-κB as well as IRF3-IRF7 dimer formation after binding of the CARDs domain of RLR to MAVS in mitochondria. The cGAS-STING also detects the dsRNA followed by induction of IRF3-IRF7 dimer formation, which increases IFNα/β gene expression. This IFNα/β activates the cytokines via the JAK-STAT pathway in an autocrine and paracrine manner. Additionally, the same IRFs and NF-κB pathway get activated by the herpes viruses as well as bacteria like *E. coli, P.aeruginosa*, and *Clostridium sp*.

SARS-CoV-2 S protein is primed by cellular serine protease TMPRSS2 and invades host cells using ACE2 receptors (Figure 2) [103]. S proteins are a trimeric class of proteins with two subunits, namely S1 and S2. S1 facilitates the attachment, and S2 is responsible for the fusion of viral protein to the host receptor [151]. S1 and S2 together have 22 N-glycosylation sites (presence of 14–16 glycans) and four O-glycosylation sites, and these sites are primarily responsible for host priming, antibody recognition, steric hindrance, innate and adaptive responses [152]. S1 subunit contains two domains N-terminal domain (NTD) and the C-terminal domain, the RBD. RBD contains highly sialylated glycans at N234 and N282, which determine viral attachment with ACE2 host receptors [153]. O-glycans are suggested to form a mucin-like domain known to shield the virus from immune invasion [154].

The pattern recognition family (PRR), which recognizes RNA, includes Toll-like receptors like the TLR 3, 7, and 8. TLR3 discriminately recognizes viruses with double-stranded RNA (dsRNA), either genome or virus replication intermediates, whereas TLR7 and 8 recognize ssRNA [155]. The cytosolic receptors for viral RNA recognition include cyclic GMP-AMP Synthase-Stimulator of interferon genes (cGAS-STING) and family of RIG-I (retinoic acid-inducible gene I)-like receptors (RLRs) and melanoma differentiation factor 5 (MDA5). The RLRs have caspase activation and recruitment domain (CARDs) and inhibitory helicase domain (Figure 2) [156]. After attaching RIG-I to the 5′ PPP end of RNA, the inhibitory helicase domain of CARDs gets removed, and it propagates to the interior of dsRNA after hydrolyzing an ATP molecule [155].

RIG-I is further activated via polyubiquitination and triggers RIG-I receptor tetramerization. RIG-1 then binds with mitochondrial antiviral signaling protein (MAVS) to set off activation of interferon regulatory factors (IRF) and NF-κB (Figure 2) [155]. NF-κB not only targets regulatory genes of inflammatory cytokines and cell survival, proliferation, and several cell surface proteins [155]. Further, the Janus Kinase-signal transducer and activator of transcription protein (JAK-STAT) pathway is activated by IFN-α/β [157]. Activation of the JAK-STAT pathway contributes to inflammation by secreting interferon-stimulated genes (ISG), 2′-5′-oligoadenylate synthetase (OAS), and IRF-7 [157,158].

Moreover, TLR7/8 detects ssRNA in the endosome and recruits a few factors like TRAF, ultimately activating interferon genes (Figure 2) [155]. Even the DNA viruses, like human papillomavirus (HPV), Epstein-Barr Virus (EBV), Kaposi's sarcoma-associated herpesvirus (KSHV), and Merkel cell polyomavirus induce TLR9 detected type I interferon (IFN) secretion [159]. These viruses also activate several PRR families like RLRs, cGAS-STING, and PYHIN protein culminating in the NF-κB pathway like SARS CoV [160, 161]. Additionally, several bacteria like *Pseudomonas aeruginosa (P. aeruginosa)*, *Escherichia coli* (*E. coli)* (gram-negative), and *Clostridium sp.* (gram-positive), are detected by TLR4 and TLR1/2/6 respectively [162, 163]. Also, these bacteria activate the same inflammatory cytokines/chemokines pathways [164]. Hence, SARS-CoV-2 possibly triggers inflammation in the lungs via NF-κB and IRFs, while these factors also get activated in several other bacterial and viral infections.

## 7 Possible and in-use diagnostics to detect SARS-CoV-2 infection: an urgent requirement

The first stepping stone to combat is to find effective diagnosis methods. We have provided an overview of various diagnostic techniques that are useful in the case of SARS-CoV-2 detection.

### 7.1 Molecular biology-based methods

Currently, quantitative reverse transcriptase PCR (qRT-PCR) is favored over conventional reverse transcriptase PCR (RT-PCR) for the detection of CoV as it is a way more sensitive technique (Table 2) [165]. Additionally, an advanced variant of PCR, the digital droplet PCR (ddPCR), has an edge over qRT-PCR. The ddPCR division of reaction mixture into tens of thousands of nanodroplets improves detection dynamics and accuracy [166].

**Table 2:** Overview of various diagnostic techniques for detection of SARS-CoV-2 infection.

Diagnostic Tests	Sample	Target	Advantages	Limitations	Ref.
**Molecular methods**					
Real time-reverse transcriptase PCR	Throat swab	Viral genetic material	• LoD∗: 3.6–3.9 copies /reaction • Detection probability: 95%.	• Requires bulky instrumentation and expensive prerequisites • Approximate assay reaction time: 120 min • Sample-to-result time: almost 4 h	[165] [177]
Digital droplet PCR	Throat swab	Viral genetic material	• Detection range: 10 to 5 x 10^4^ copies/reaction • Sensitivity: 94% • LoD∗: 10 copies/test	• Expensive • Need experts to perform the test	[166]
CRISPR-Cas12 based method	Nasal and oro-pharyngeal swab	Viral genetic material	• Analytical LoD: 10 copies/ul input • Portable • Approximate sample-to-result assay time: 45 min.	• Qualitative output • Expensive • Need expert to perform	[167]
**Serological test**					
ELISA	Blood	Virus specific Antibody	• Sensitivity: 74.3–77.1% • Accuracy: 97.3% • Can identify multiple pathogens at a single time	• Primary disease diagnosis is not possible as 30–50% of positive rate observed at 0–10 d.p.o∗∗ • Invasive	. [178]
IgM-IgG combined detection	Blood	Virus specific antibody	• Sensitivity: 88.6% • Specificity: 90.63% • Minimize the chances of false positive test	• Time consuming • Labour intensive • Invasive method	[107]
**Radiological tests**					
Chest CT	Lung scan	Lungs morphological and patho-physiological changes	• Non-invasive • Less labour-intensive process • Identification of ground-glass opacification and consolidation with interlobular septum thickening as well as paving pattern	• Not-specific the infection type • Huge and non-portable machines required for examination. • Involves exposure to radiations • Need experts to decipher the scans	[179] [180]

(∗LoD = Limit of detection, ∗∗d.p.o = days post infection).

Also, clustered regularly interspaced short palindromic repeats (CRISPR) based detection methods can be used for the detection of SARS-CoV-2. Here, simultaneous, reverse transcription with isothermal loop-mediated amplification is carried out, followed by cas-12 screening for RNA extracted from patient samples [167]. Furthermore, CRISPR-based specific high-sensitivity enzymatic reporter unlocking (SHERLOCK) method, combined with isothermal loop-mediated amplification [168], can be a promising SARS-CoV-2 detection technique [169]. It includes cas-12/cas-13 mediated detection via fluorescence and colorimetric readouts (Table 2) [170]. However, CRISPR based SHERLOCK technology developed for the detection of SARS-CoV-2 remains to be verified and tested using clinical samples.

### 7.2 Serological test

Also, with rapid IgG/IgM detection through immunoassay, Enzyme-linked immunosorbent assay (ELISA) can be used as a detection method of SARS-CoV-2 infection (Table 2) [107, 171, 172]. The nucleocapsid and spike protein-based ELISA can be used for SARS-CoV-2 detection [40].

### 7.3 Chest computed axial tomography (CT) and clinical methods

Being a non-invasive, time saving, and less labor-intensive process, chest CT is the most favored diagnostic method [173]. Also, liver function tests could indicate SARS-CoV-2 infection as altered liver function is observed in COVID-19 patients [174]. This includes changed levels of different liver enzymes, a predominance of monocytes in sputum, high level of activated prothrombin, bilirubin, troponin, decrease in pro-albumin, and albumin [175, 176]. By balancing the pros and cons of the methods, more economical and optimal options can be obtained.

## 8 Drugs and vaccines for SARS-CoV-2

Researchers and pharma industries are working relentlessly for the development of new drugs and vaccines against SARS-CoV-2. Further, considering the urgency and time taken to develop new drugs or vaccines, different regulatory bodies have relaxed the norms for development and trials [181]. WHO has also launched a "Solidarity" clinical trial for COVID-19 treatments, to reduce the time taken by randomized clinical trials by 80% [182].

Moreover, several studies are going on for repurposing of the existing drugs against COVID-19, including the Solidarity trial, which involves antiviral drugs like Remdesivir, Lopinavir, Ritonavir, anti-malarial drugs like Chloroquine and Hydroxychloroquine and drugs used in the treatment of multiple sclerosis, i.e., IFNβ-1a [183]. Furthermore, medications like Baricitinib [184], Galidesivir [185, 186], Ribavirin [187, 188], Azithromycin, a common antibiotic [189] are some of the drugs which are repurposed for the COVID-19 treatment. With some studies supporting the effectiveness of these drugs and some disagreeing with it, these drugs need to be used cautiously and subjected to detailed research.

Preventing a disease is always better than treating it. Different companies and research groups have already taken the initiative to develop vaccines against COVID-19. Moreover, they are in various development phases, from preclinical stages to clinical trials; some of the promising vaccines already in the clinical phase are listed in Table 3. Further, about 150 vaccine candidates are in different stages of pre-clinical phase [190].

**Table 3:** Vaccine candidates from different organizations, which are in various phases of clinical trials.

Type	Organization	Constituents	Phase of develop-ment
Whole Virus Vaccines	Wuhan Institute of Biological Products/Sinopharm	Inactivated SARS-CoV-2	3
	Beijing Institute of Biological Products/Sinopharm	Inactivated SARS-CoV-2	3
	Sinovac	Inactivated SARS-CoV-2 + alum	3
	Institute of Medical Biology, Chinese Academy of Medical Sciences	Inactivated SARS-CoV-2	2
	Research Institute for Biological Safety Problems, Rep of Kazakhstan	Inactivated SARS-CoV-2	1/2
	Bharat Biotech	Whole virion inactivated	1/2
Nucleic acid vaccines	BioNTech/Fosun Pharma/Pfizer	3 LNP-mRNAs	3
	Inovio Pharmaceuticals/International Vaccine Institute	DNA plasmid vaccine with electroporation	1/2
	Moderna/NIAID	LNP encapsulated mRNA	1
	Genexine Consortium	DNA Vaccine (GX-19)	1/2
	Cadila Healthcare Limited	DNA plasmid vaccine	1/2
	Osaka University/AnGes/Takara Bio	DNA plasmid vaccine + Adjuvant	1/2
	Imperial College London	LNP-nCoVsaRNA	1
	Curevac	mRNA	2
	People's Liberation Army (PLA) Academy of Military Sciences/Walvax Biotech	mRNA	1
	Arcturus/Duke-NUS	mRNA	1/2
Non-replicating viral vector	University of Oxford/AstraZeneca	ChAdOx1-S	3
	CanSino Biological Inc./Beijing Institute of Biotechnology	Adenovirus Type 5 Vector	2
	Gamaleya Research Institute	Adeno-based	3
	Janssen Pharmaceutical Companies	Ad26COVS1	3
	ReiThera/LEUKOCARE/Univercells	Replication defective Simian Adenovirus (GRAd) encoding S	1
	Institute of Biotechnology, Academy of Military Medical Sciences, PLA of China	Ad5-nCoV	1
Replicating viral vector	Institute Pasteur/Themis/Univ. of Pittsburg CVR/Merck Sharp & Dohme	Measles-vector based	1
	Beijing Wantai Biological Pharmacy/Xiamen University	Intranasal flu-based-RBD	1
Protein Subunit	Novavax	Full length recombinant SARS CoV-2 glycoprotein nanoparticle vaccine adjuvanted with Matrix M	2b
	Clover Biopharmaceuticals Inc./GSK/Dynavax	Native like Trimeric subunit Spike Protein vaccine	1
	Anhui Zhifei Longcom Biopharmaceutical/Institute of Microbiology, Chinese Academy of Sciences	Adjuvanted recombinant protein (RBD Dimer)	2
	Vaxine Pty Ltd/Medytox	Recombinant S protein with Advax™ adjuvant	1
	University of Queensland/CSL/Seqirus	Molecular clamp stabilized Spike protein with MF59 adjuvant	1
	Medigen Vaccine Biologics Corporation/NIAID/Dynavax	S–2P protein + CpG 1018	1
	Instituto Finlay de Vacunas, Cuba	RBD + Adjuvant	1
	FBRI SRC VB VECTOR, Rospotrebnadzor, Koltsovo	Peptide	1
	West China Hospital, Sichuan University	RBD (baculovirus production expressed in Sf9 cells)	1
	University Hospital Tuebingen	SARS-CoV-2 HLA-DR peptides	1
	COVAXX	S1-RBD-protein	1
	Sanofi Pasteur/GSK	S protein (baculovirus production)	2
	Kentucky Bioprocessing, Inc	RBD based	2
VLP	Medicago Inc./Université Laval	Plant-derived VLP	1

(Adapted from- WHO Draft landscape of COVID-19 candidate vaccines – 22^nd^ Sept 2020, with modifications).

## 9 Future directions in COVID-19 research

The previous epidemics like plague, smallpox, cholera, ebola, SARS, and many others have extended the horizon of human knowledge on pathogenic infectious diseases and compelled us to acquire new weapons for fighting and eradicate these. The current COVID-19 pandemic needs to be addressed rapidly but with patience. Moreover, in the fight against this deadly disease, past experiences and acquired knowledge about SARS-CoV-2 to date will help shape the future of diagnosis, treatment, and prevention. Diagnosis or prognosis is the gateway to fighting any disease; the degree of success largely depends on the correct and on-time diagnosis. Various molecular, biophysical, immunological, and biochemical diagnostic methods are used for the detection of the SARS-CoV-2 infection [170, 191]. Moreover, qRT-PCR is the most trusted and immune or antibody-based detection is useful for large scale screening [165]. Additionally, a CT scan is more effective in predicting disease severity [173].

One of the crucial aspects to be taken care of during the treatment of COVID-19 is the associated comorbidities. As people suffering from heart diseases, hypertension, and diabetes may be negatively impacted by ACEIs and ARB inhibitors for COVID-19 treatment. Furthermore, drugs like chloroquine, hydroxychloroquine, remdesivir, and tocilizumab recommended for SARS-CoV-2 infection, can cause irregularities in the arteries, kidney, liver, and nervous system [192]. Hence people, who have one or more comorbidities or have immunosuppressed conditions should be treated with caution, else can cause more fatality [193].

In the current situation, drug repurposing is one of the safest options to be considered and implemented to treat the pandemic. Moreover, as these drugs are short term majors, new drugs and vaccines need to be developed for avoiding future consequences. However, the standard development process should be followed with different vaccines as a better alternative or as a backup if the current one is entirely or partially ineffective. For the time being, as an alternative to vaccines or nonspecific drugs, plasma infusion therapy can be used as in other diseases [194]. Also, in this respect, plants can serve as sources of various antiviral substances like plant lectins, which are proven to be effective against Human Immunodeficiency Virus [195]. Various *in-silico* studies have suggested that plants' active compounds such as withaferin A, withanolide D, quercetin, epigallocatechin gallate (EGCG), and hypericin have the potential to inhibit the transmission of SARS-CoV-2 infection [196, 197]. The effect of withanone on TMPRSS2 expression in MCF7 cells is examined and found to remarkably downregulated TMPRSS2 mRNA in treated cells. This predicts the dual action of withanone to block SARS-CoV-2 entry into the host cells [192]. Interestingly, EGCG is under phase-2 clinical trial for COVID-19 disease [198].

Also, as these viruses have a zoonotic origin, consumption of meat from close relative animals should be avoided. The government should take measures to prevent selling multiple meat varieties together to minimize the chances of cross-species virus transmission. Additionally, the viruses that can be virulent and causes such an epidemic needs to be studied in detail to avoid this kind of devastating situation in the future.

## 10 Conclusion and limitations

SARS-CoV-2 has an immense potential to infect millions across the globe. The virus can undergo mutations in several of its proteins, like the S protein, N protein, PLpro, and ORF3a, making it more or less suitable or virulent to humans. Additionally, many computational and phylogenetic analyses have been done to explain the mutation in these proteins and their relativity to various human pathogenic viruses. This, in turn, could shed light on the virus's ability to bind to host surfaces, manipulate host cell functions, etc. However, *in vitro* and *in vivo* experimental studies establishing these facts remain to be conducted. Numerous clinical reports suggest the association of various comorbidities like diabetes, hypertension, cardiovascular diseases, and related immunocompromised situations to COVID-19. However, no particular treatment against COVID-19 is available, keeping in view the patient's comorbid condition. Certain pathophysiological changes in the lungs and the alterations at the cellular and molecular level post-SARS-CoV-2 infection are also reported. A clear distinction of these pathologies exclusive to COVID-19 does not exist; most of these pathologies are similar to those induced by viruses like SARS or MERS. Furthermore, experimental analysis corroborating the speculations and findings of virus-mediated host cell responses initiated by signaling molecules, like NF-κB, IRFs remains a subject of investigation. Finally, a continuous upgrade in the diagnostic system is necessary for better diagnosis and prognosis of the disease to reduce the rate of mortality. Rigorous research regarding SARS-CoV-2 induced COVID-19 is an urgent need of the hour. Conclusively, addressing the above limitations requires prior information on various aspects of COVID-19, covered in our review article. Our review will help the scientific community by providing necessary information about different aspects of the current pandemic on a single platter.

## Declarations

### Author contribution statement

All authors listed have significantly contributed to the development and the writing of this article.

### Funding statement

This work was supported by 10.13039/501100001332Council for Scientific and Industrial Research grant no 37(1693)/17/EMR-II, Department of Science and Technology as Ramanujan fellowship grant no. SB/S2/RJN-132/20/5. We are thankful to The Ministry of Human Resource, Department of Biotechnology, University Grants Commission, and DST-inspire for fellowship to Shweta Jakhmola, Omkar Indari, Budhadev Baral and Nidhi Varshney respectively and CSIR for a scholarship to Charu Sonkar, Dharmendra Kashyap, and Annu Rani in the form of research stipend.

### Data availability statement

Data included in article.

### Competing interest statement

The authors declare no conflict of interest.

### Additional information

No additional information is available for this paper.
